# Supplementary material for: 3D printed microchannels for sub-nL NMR spectroscopy
Source: PLoS One. 2018 May 9;13(5):e0192780. doi: 10.1371/journal.pone.0192780 (PMC5942786; doi:10.1371/journal.pone.0192780)
Supplement: S1 File — Fig A. Sensitivity map for the NMR microcoil computed via a Biot-Savart based code implemented in Matlab. For the NMR microcoil, the local sensitivity is defined as Buxy(r)sin(γB1xy(r)τ). The coil is in the yz plane and the static magnetic field B0 is along the z axis. Sensitivity distribution in the xz (a and c) and xy (b and d) planes for NMR coil with excitation parameters τ = 3.7 μs and I = 9 mA. The dashed lines indicate the position of the Rc ovum (a and b) and the C. elegans worm (c and d). Fig B. Illustration of the fluidic interface. (a) The microfluidic channel is attached to a PMMA rod and connected to PMMA capillaries to create a fluidic assembly. (b) The fluidic assembly is mounted in the holder. (c) The Micro-to-macro interface is completed by connecting tubes to the PMMA capillaries and performing the casting of epoxy resin to create the sealing and give robustness to the fluidic system. Fig C. Schematic of the fluidic set-up used for the loading and trapping of biological samples. (1) Microfluidic chip and its fluidic interface. (2) Inverted microscope (Axio Observer, Zeiss, Germany), High-Power LED Illumination system (precisExcite, Visitron, Germany) for brightfield imaging. (3) Liquid waste. (4) High resolution digital camera (ORCA-ER C4742-80, Hamamatsu, Japan). (5) Syringe pumps (Nemesys, Cetoni GmbH, Germany). (6) Desktop computer. Fig D. Schematic of the electronics setup used for the NMR measurements. (1) Integrated excitation/detection coil interfaced with the 3D printed microchannel through a micro-to-macro fluidic interface. (2) Single chip NMR detector (see details in Ref. [72]). (3) Printed circuit board (PCB). (4) Superconductive magnet (Bruker, 7 T). (5) RF source (MG3633A, Anritsu; Japan). (6) AF amplifier (SRS560, Stanford Research Systems, USA) (7) Multifunctional board (PCIe-6259, National Instruments, USA) for the generation of Tx/Rx switching pulse and signal acquisition. Fig E. 1H spectrum of H2O in the Rc ovum-dedicated microch [file pone.0192780.s001.docx]

3D printed microchannels for sub-nL NMR spectroscopy

E. Montinaro^1^, M. Grisi^1^, M. C. Letizia^1^, L. Pethö^2^, M. A. M. Gijs^1^, R. Guidetti^3^, J. Michler^2^, J. Brugger^1^, and G. Boero^1,*^

*^1^Ecole Polytechnique Fédérale de Lausanne (EPFL), Laboratory for Microsystems, Lausanne, Switzerland*

*^2^Swiss Federal Laboratories for Materials Science and Technology (EMPA), Laboratory for Mechanics of Materials and Nanostructures,, Thun, Switzerland*

*^3^University of Modena and Reggio Emilia, Department of Life Sciences, Modena, Italy*

**Corrisponding author*

*E-mail:* [*giovanni.boero@epfl.ch*](mailto:giovanni.boero@epfl.ch) *(GB)*

Section A: Maps of sensitivity


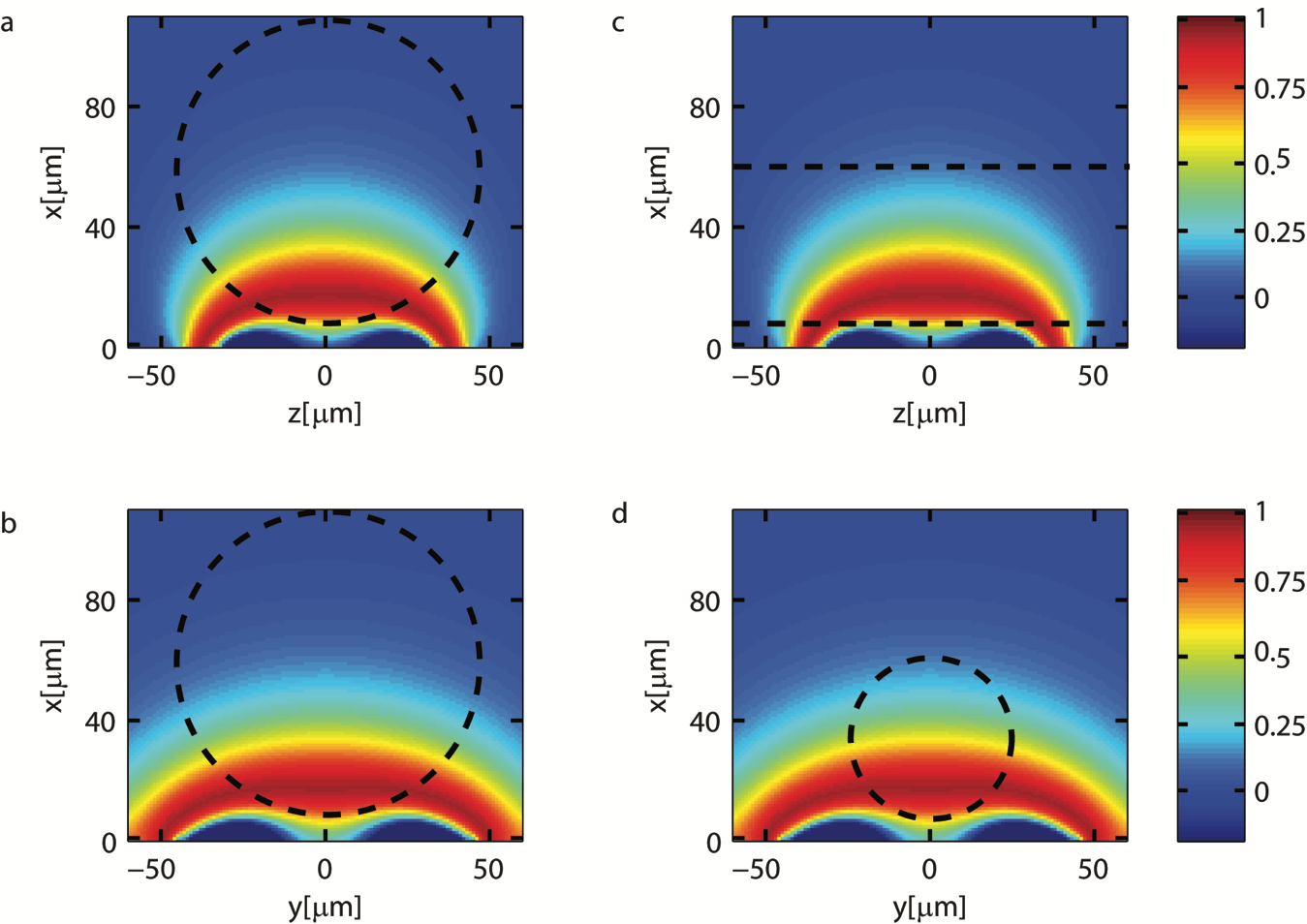
The signal contribution (*i.e* local sensitivity) *dS(****r****)* per elementary volume *dV_S_* is proportional to *B_uxy_(****r****)sin(Ɵ).* This dependence is used to evaluate the sensitivity of the coil, starting from the computation of *B_uxy_(****r****)* via a Biot-Savart based code implemented in Matlab. The coil is in the *yz* plane and the static magnetic field B_0_ is along the *z* axis. Fig A shows maps of sensitivities of the NMR integrated coil in the *xz* (Fig Aa and Fig Ac) and in the xy (Fig Ab and Fig Ad) planes. The dashed lines indicate the position in space of the *Rc* ovum (Fig Aa and Fig Ab) and the *C. elegans* worm (Fig Ac and Fig Ad) trapped in the microchannels and aligned with the integrated microcoil.

Fig A. Sensitivity map for the NMR microcoil computed via a Biot-Savart based code implemented in Matlab. For the NMR microcoil, the local sensitivity is defined as *B_uxy_*(r)sin(*γB_1xy_*(r)*τ*)*.* The coil is in the *yz* plane and the static magnetic field *B_0_* is along the *z* axis. Sensitivity distribution in the *xz* (a and c) and *xy* (b and d) planes for NMR coil with excitation parameters *τ* = 3.7 µs and *I* = 9 mA. The dashed lines indicate the position of the *Rc* ovum (a and b) and the *C. elegans* worm (c and d).

Section B: Fluidic interface


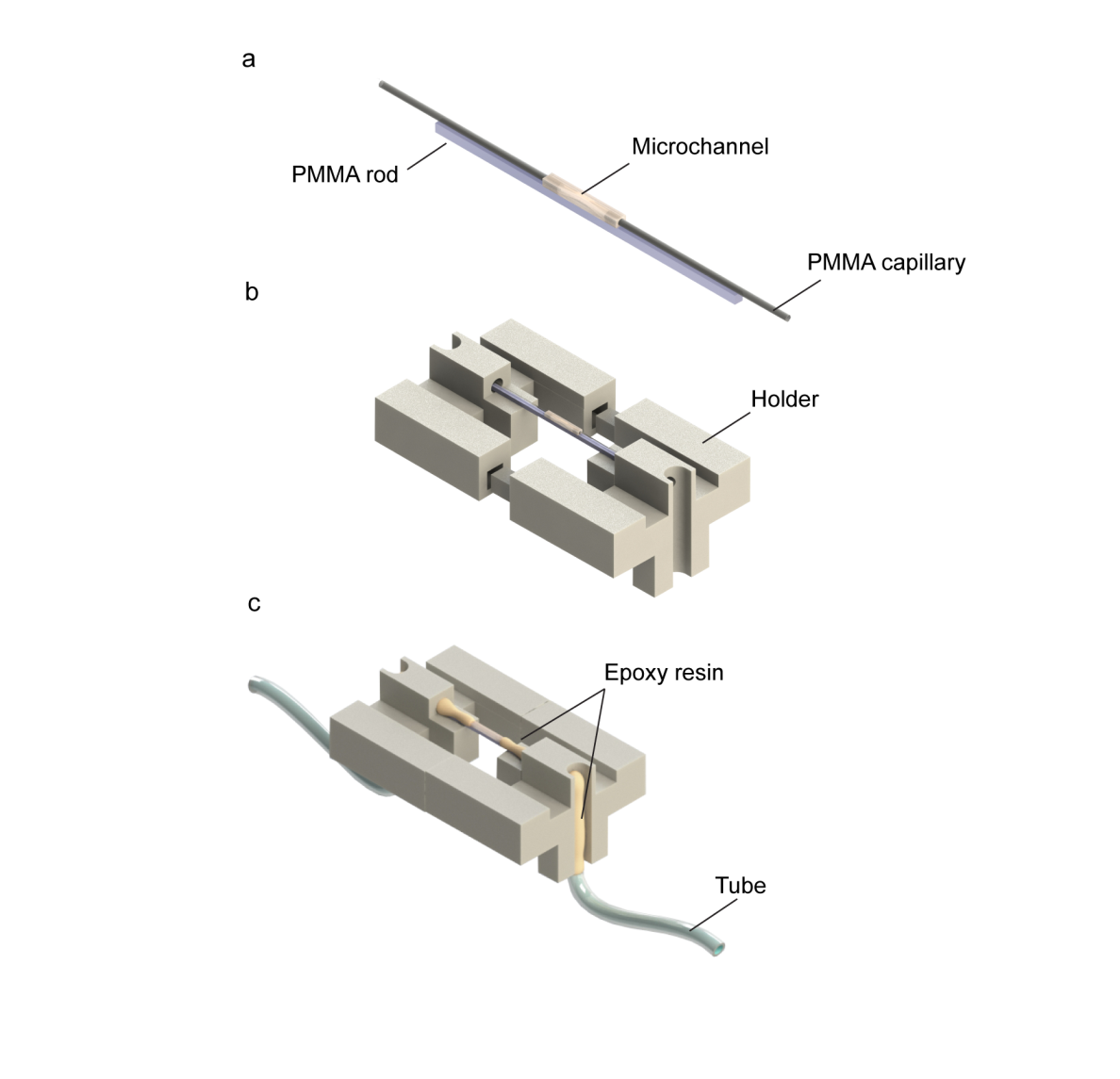
In order to precisely position and tightly hold the high resolution 3D printed microfluidic chips in contact with the single-chip integrated CMOS detector (and to connect its microchannels to the external pump) an interfacing structure is fabricated. The 3D printed microfluidic chips are first glued on a 200 µm x 2 cm PMMA rod for support using a cyanoacrylate adhesive (ECS500, 3M). After, PMMA capillaries (Paradigm Optics, USA) are fitted in the inlet and the outlet of the 3D printed microchannels. Finally, the realized assembly shown in Fig Ba is introduced into a 3D printed plastic holder (Fig Bb) fabricated by a conventional stereolithographic 3D printer (Form+1, Formlabs, USA). The holder is patterned out of a photosensitive resin (Clear FLGPCL02, Formlabs, USA) and is constituted of two complementary parts to allow for the insertion of the fluidic assembly. A system of tubes is connected to the fluidic assembly through the PMMA capillaries and a casting of epoxy resin (Araldite) is performed to seal the fluidic system and to give robustness to the structure. The assembled fluidic interface (Fig Bc) is manually brought into contact and aligned to the integrated excitation/detection microcoil under an optical microscope. Wax is used to maintain the interface in position. A central squared aperture in the holder provides visibility for the alignment. The printed circuit board (PCB), containing the single-chip integrated NMR detector, includes two elongated holes to allow for sufficient free planar movement for the microchannel-to-microcoil alignment. With this system, we repeatedly achieved a good seal, easy handling of the liquids, and a positioning of the sample within 10 μm precision.

**Fig B. Illustration of the fluidic interface.** (**a**) The microfluidic channel is attached to a PMMA rod and connected to PMMA capillaries to create a fluidic assembly. (**b**) The fluidic assembly is mounted in the holder. (**c**) The Micro-to-macro interface is completed by connecting tubes to the PMMA capillaries and performing the casting of epoxy resin to create the sealing and give robustness to the fluidic system.

Section C: Fluidic set-up for sample loading and trapping

Fig C shows an illustration of the fluidic set-up. The 3D printed microfluidic chip (together with its fluidic interface) is placed into an inverted microscope (Axio Observer, Zeiss, Germany) equipped with a High-Power LED Illumination system (precisExcite, Visitron, Germany) for brightfield imaging. The microscope is equipped with a motorized *xyz* stage that has a piezo controller for *z* displacement (ASI, Visitron, Germany). The microfluidic operations are controlled using syringe pumps and its software (Nemesys, Cetoni GmbH, Germany).

**Fig C. Schematic of the fluidic set-up used for the loading and trapping of biological samples.** (**1**) Microfluidic chip and its fluidic interface. (**2**) Inverted microscope (Axio Observer, Zeiss, Germany), High-Power LED Illumination system (precisExcite, Visitron, Germany) for brightfield imaging. (**3**) Liquid waste. (**4**) High resolution digital camera (ORCA-ER C4742-80, Hamamatsu, Japan). (**5**) Syringe pumps (Nemesys, Cetoni GmbH, Germany). (**6**) Desktop computer.

Section D: NMR set-up

Fig D shows an illustration of the NMR experimental set-up. The single-chip CMOS integrated NMR detector is glued onto a printed circuit board (PCB) and electrically connected by wire bonding. A single RF generator is used to provide the RF signal for the transmission (Tx) and as local oscillator (LO) for the on-chip frequency down-conversion. The frequency down-converted and amplified signal and the output of the single-chip NMR detector is further amplified by an external amplifier and sent to a multifunctional board for acquisition. The PCB is inserted in the 54 mm room temperature bore of a 7.05 T superconducting magnet (Bruker, 300 MHz). The 3D printed microchannel lies along the direction of the static magnetic field to reduce the effect of susceptibility mismatches. In this configuration, the NMR linewidth is about a factor of two narrower with respect to the configuration where the channel is perpendicular to the static magnetic field.

**Fig D. Schematic of the electronics setup used for the NMR measurements**. (**1**) Integrated excitation/detection coil interfaced with the 3D printed microchannel through a micro-to-macro fluidic interface. (**2**) Single chip NMR detector (see details in Ref. ([1](#_ENREF_1))). (**3**) Printed circuit board (PCB). (**4**) Superconductive magnet (Bruker, 7 T). (**5**) RF source (MG3633A, Anritsu; Japan). (**6**) AF amplifier (SRS560, Stanford Research Systems, USA) (**7**) Multifunctional board (PCIe-6259, National Instruments, USA) for the generation of Tx/Rx switching pulse and signal acquisition.

Section E: ^1^H NMR spectrum of H_2_O in a microchannel designed for ovum trapping

Fig E shows the ^1^H NMR spectrum of water after averaging over 2 hours. The NMR experiment is performed using the 3D printed microchannel designed for the handling and the trapping of a single *Rc* ovum, interfaced with the holding structure described in section A. The chemical shift is expressed in ppm, assigning a chemical shift of 4.8 ppm to the peak of water. The excitation pulse length (3.5 µs) used in the reported experiments corresponds to the experimental condition of maximum signal-to-noise ratio. The spectrum is obtained at 7.05 T (300 MHz) and is normalized to the amplitude of the peak of water. The linewidth, defined as spectral peak width at half-maximum and determined through the fit of a Lorentzian curve to the data, is equal to 10 Hz FWHM. The baseline width, defined as the peak width at 0.55% height of the peak of water, is equal to 75 Hz.

**Fig E. ^1^H spectrum of H_2_O in the *Rc* ovum-dedicated microchannels, performed at 7.05 T.** The spectra are the real parts of the Fast Fourier Transform (FFT) of the time-domain NMR signals. Notations: *V* is the active volume, *Avg* number of averaged measurements, *T*_R_ is the repetition time, *τ* is the pulse length, *T*_m_ is the matching filter decay time constant. *V ≅ 250* pL, *Avg* = 1800, *T*_R_ = 2s, *τ* = 3.5 µs, *T*_m_ = ∞.

Section F: Optimization of the repetition time

Figure F shows the ^1^H NMR spectra obtained from a single *Rc* ovum in H_2_O, with a repetition time $T_{R}$ of 2 s (Fig Fa), 200 ms (Fig Fb) and 50 ms (Fig Fc) after averaging over 12 hours, all obtained with the same pulse length τ = 3.5 µs. This pulse length maximize the amplitude of the signal at 1.3 ppm for $T_{R}$ = 2 s and longer. For all spectra the signal amplitude is normalized to the amplitude of the peak at 1.3 ppm obtained with a $T_{R}$ of 2 s. Reducing the repetition time *T*_R_ from 2 s to 200 ms, the amplitudes of all observed peaks, water included, do not change significantly (in our previous study of the *Rc* ova,([2](#_ENREF_2)) we used a non-optimum repetition time of 2 s). Such faster repetition rate allow for a reduction of the experimental time of a factor 3.3 for the same SNR. A detailed study of the spin-lattice (and spin-spin) relaxation times of the ^1^H nuclei contained inside the different compounds in the investigated biological samples will be reported elsewhere. Due to the weak signal amplitudes and the highly inhomogeneous RF excitation field *B_1_*, these investigations requires very long averaging time and computational care.

**Fig F.** **NMR measurements of a single *Rc* tardigrade ovum in H_2_O at different repetition times.** NMR measurements performed at 7 T. See definition of notation in Fig. S5. (**a**): *V* ≅ 210 pL; *Avg* = 21600; *T*_R_ = 2 s, *τ* = 3.5 µs, *T*_m_ = 30 ms. (**b**): *V* ≅ 210 pL; *Avg* = 216000; *T*_R_ = 200 ms, *τ* = 3.5 µs, *T*_m_ = 30 ms. (**c**): *V* ≅ 210 pL; *Avg* = 864000; *T*_R_ = 50 ms, τ = 3.5 µs, *T*_m_ = 30 ms.

Section G: ^1^H NMR spectrum of *E.coli* in S medium

Figure G shows the ^1^H NMR spectrum of *E. coli* in S medium (prepared following the protocol reported in Ref. ([3](#_ENREF_3))) at a concentration of 1.5 x 10^9^ cell/mL, obtained after 11 hours of averaging. This solution is used to feed the *C. elegans*. The signal acquired from the *E. coli* solution, ingested by the worm during the experiment, may contribute to the NMR spectrum obtained from the *C. elegans* subsection. All chemical shifts are expressed in ppm deviation from the resonance frequency of tetramethylsilane (TMS). Since this reference compound is not present in our samples, we assigned a chemical shift of 4.8 ppm to the peak of water, thus determining the chemical shifts of the other peaks. The excitation pulse length (3.5 µs) corresponds to the experimental condition of maximum signal-to-noise ratio. The spectrum is obtained using the 3D printed microchannel designed for the trapping of a single *C.* *elegans* and it is normalized to the amplitude of the peak of water.

**Fig G.** **^1^H spectrum of *E.coli* in S medium.** NMR measurement performed at 7 T, using the 3D oriented microchannel designed for the trapping of a single *C.* *elegans*. See definition of notation in Fig S4. *V* ≅ 100 pL; *Avg* = 19800; *T_R_* = 2 s, *τ* = 3.5 µs, *T_m_* = 60 ms.

References

1. Grisi M, Gualco G, Boero G. A broadband single-chip transceiver for multi-nuclear NMR probes. Rev Sci Instrum. 2015;86(4):044703.

2. Grisi M, Vincent F, Volpe B, Guidetti R, Harris N, Beck A, et al. NMR spectroscopy of single sub-nL ova with inductive ultra-compact single-chip probes. Scientific Reports. 2017 Mar 20;7. PubMed PMID: WOS:000396667400001. English.

3. Stiernagle T. Maintenance of C. elegans. C elegans. 1999;2:51-67.
